# Optimization and evaluation of Flexicult^®^ Vet for detection, identification and antimicrobial susceptibility testing of bacterial uropathogens in small animal veterinary practice

**DOI:** 10.1186/s13028-015-0165-4

**Published:** 2015-10-26

**Authors:** Luca Guardabassi, Sandra Hedberg, Lisbeth Rem Jessen, Peter Damborg

**Affiliations:** Department of Veterinary Disease Biology, Faculty of Health and Medical Sciences, University of Copenhagen, Stigbøjlen 4, 1870 Frederiksberg C, Denmark; Department of Veterinary Clinical and Animal Sciences, Faculty of Health and Medical Sciences, University of Copenhagen, Dyrlægevej 16, 1870 Frederiksberg C, Denmark

**Keywords:** Urinary tract infections, Dogs, Cats, On-site diagnostics, Antimicrobial stewardship

## Abstract

**Background:**

Urinary tract infection (UTI) is a common reason for antimicrobial prescription in dogs and cats. The objective of this study was to optimize and evaluate a culture-based point-of-care test for detection, identification and antimicrobial susceptibility testing of bacterial uro-pathogens in veterinary practice.

**Methods:**

Seventy-two urine samples from dogs and cats with suspected UTI presenting to seven veterinary facilities were used by clinical staff and an investigator to estimate sensitivity and specificity of Flexicult Vet A compared to laboratory reference standards for culture and susceptibility testing. Subsequently, the test was modified by inclusion of an oxacillin-containing compartment for detection of methicillin-resistant staphylococci. The performance of the modified product (Flexicult Vet B) for susceptibility testing was evaluated in vitro using a collection of 110 clinical isolates.

**Results:**

Bacteriuria was reported by the laboratory in 25 (35 %) samples from the field study. The sensitivity and specificity of Flexicult Vet A for detection of bacteriuria were 83 and 100 %, respectively. Bacterial species were correctly identified in 53 and 100 % of the positive samples by clinical staff and the investigator, respectively. The susceptibility results were interpreted correctly by clinical staff for 70 % of the 94 drug-strain combinations. Higher percentages of correct interpretation were observed when the results were interpreted by the investigator in both the field (76 %) and the in vitro study (94 %). The most frequent errors were false resistance to β-lactams (ampicillin, amoxicillin-clavulanate and cephalotin) in *Escherichia coli* for Flexicult Vet A, and false amoxicillin-clavulanate resistance in *E. coli* and false ampicillin susceptibility in *Staphylococcus pseudintermedius* for Flexicult Vet B. The latter error can be prevented by categorizing staphylococcal strains growing in the oxacillin compartment as resistant to all β-lactams.

**Conclusions:**

Despite the shortcomings regarding species identification by clinical staff and β-lactam susceptibility testing of *E. coli*, Flexicult Vet B (commercial name Flexicult^®^ Vet) is a time- and cost-effective point-of-care test to guide antimicrobial choice and facilitate implementation of antimicrobial use guidelines for treatment of UTIs in small animals, provided that clinical staff is adequately trained to interpret the results and that clinics meet minimum standards to operate in-house culture.

## Background

Urinary tract infection (UTI) is a common reason for antimicrobial prescription in small animal veterinary practice. It has been estimated that 14 % of all dogs are diagnosed with bacterial UTI during their lifetime [1]. UTI is less frequent and usually associated with comorbidities in cats [2]. *Escherichia coli* is by far the most frequent species involved in at least 50 % of canine and feline UTIs [2, 3]. Other relatively common species include *Staphylococcus* spp., *Enterococcus* spp., *Proteus* spp., *Klebsiella* spp., *Pseudomonas* spp., and *Streptococcus* spp. [3–5]. The diagnosis is based on clinical signs, urinalysis and quantitative microbiology. Empirical antimicrobial therapy is frequent, especially in first-time uncomplicated UTIs. According to international and Danish guidelines for antimicrobial use in companion animals [6, 7], urine samples from small animals with suspected UTI should be subjected to culture and susceptibility testing when treating with antibiotics. However, several factors influence negatively implementation of this recommendation in veterinary practice, i.e. long laboratory turnaround time, special requirements for shipping (i.e. urine samples should be refrigerated if they cannot be processed within 24 h after collection), and economic costs for pet owners. In-house culture is a possible alternative to laboratory analysis but not all clinics are equipped to perform bacterial isolation and antimicrobial susceptibility testing according to international quality and biosafety standards. Even if performed optimally, in-house diagnostic culture requires at least 2 days before treatment can be initiated or adjusted based on antimicrobial susceptibility results.

Point-of-care testing is a possible approach to reduce both turnaround time and costs. Flexicult™ (SSI Diagnostica, Hillerød, Denmark) is a diagnostic product widely used in Denmark for point-of-care diagnosis and susceptibility testing of uncomplicated urinary tract infections (UTIs) in human primary health care [8]. The test allows (1) semi-quantitative enumeration of bacteria in urine, (2) presumptive identification of uropathogens and (3) prediction of antimicrobial susceptibility of uropathogens. A veterinary derivative product (Flexicult^®^ Vet), hereinafter referred to as Flexicult Vet A, was first launched in the US in December 2010. The agar content of the product for human use was modified to enhance growth of veterinary pathogens including *Staphylococcus pseudintermedius* and *Streptococcus canis*, which grow poorly on Flexicult™ plates for human use. The objective of this study was to optimize and evaluate this point-of-care test for use in small animal practice. On-site performance of Flexicult Vet A was evaluated by a field trial conducted in seven small animal veterinary clinics in Denmark. Subsequently the composition of the antimicrobial panel was optimized to enable detection of methicillin-resistant *S. pseudintermedius* (MRSP), which are multidrug-resistant bacteria of high concern in small animal veterinary practice [9]. The modified test, hereinafter referred to as Flexicult Vet B, was evaluated in vitro using a strain collection representative of all major bacterial species implicated in canine and feline UTIs.

## Methods

### Description of Flexicult Vet A

**Fig. 1:**
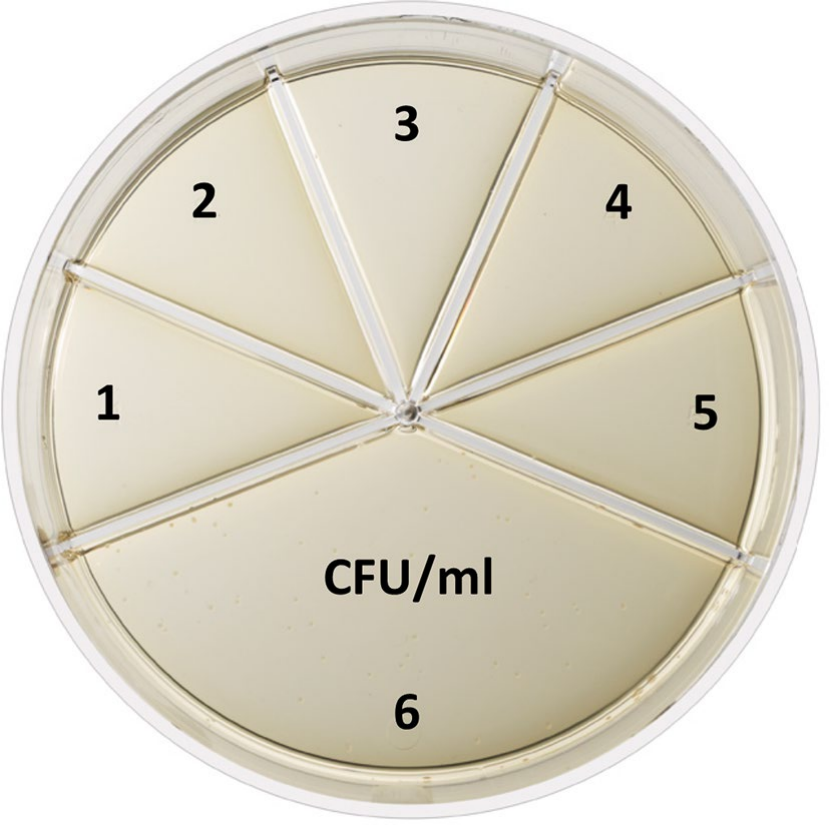
Description of Flexicult^®^ Vet. Flexicult^®^ Vet (Flexicult Vet B in the text) is a point-of-care test allowing (a) semi-quantitative enumeration of bacteria in urine, (b) presumptive identification of uropathogens, and (c) prediction of antibiotic susceptibility of uropathogens. The test consists of a plastic Petri dish divided into five smaller compartments for susceptibility testing of ampicillin (*1*), amoxicillin-clavulanate (*2*), oxacillin (*3*), enrofloxacin (*4*), and sulfamethoxazole-trimethoprim (*5*), and one large compartment containing non-selective growth medium for semi-quantitative bacterial enumeration (*6*)

**Fig. 2:**
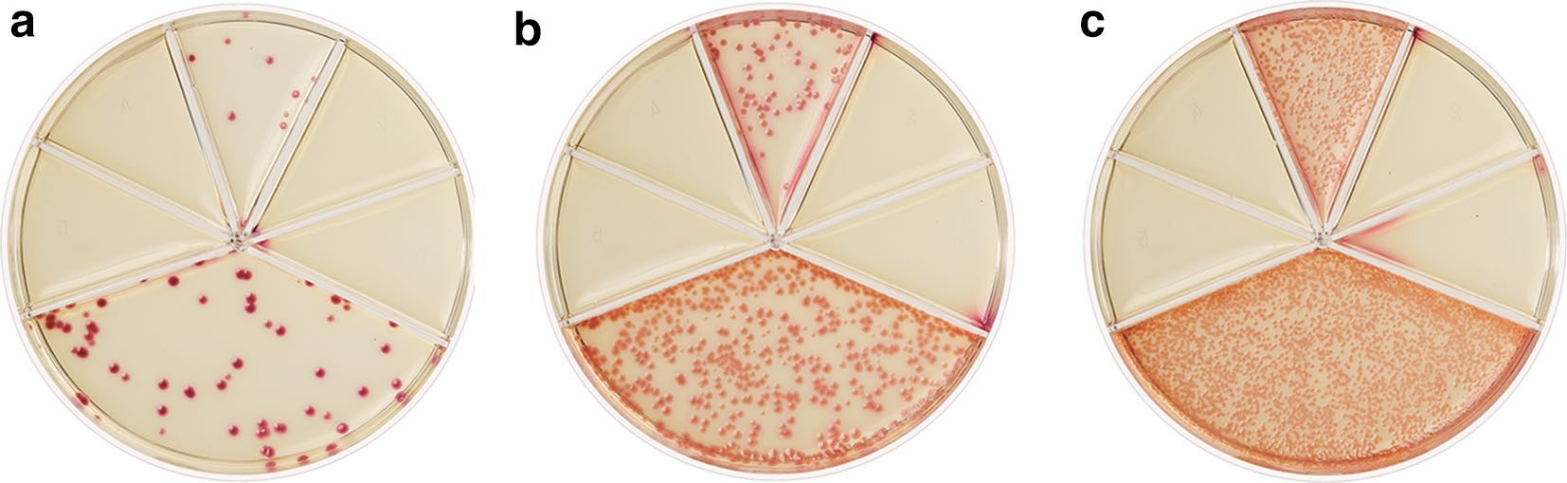
Semi-quantitative bacterial enumeration by Flexicult^®^ Vet. Growth of *Escherichia coli* at different concentrations: 10^3^ CFU/ml (**a**), 10^4^ CFU/ml (**b**) and 10^5^ CFU/ml (**c**). The strain is susceptible to all antimicrobial agents tested except oxacillin

**Fig. 3:**
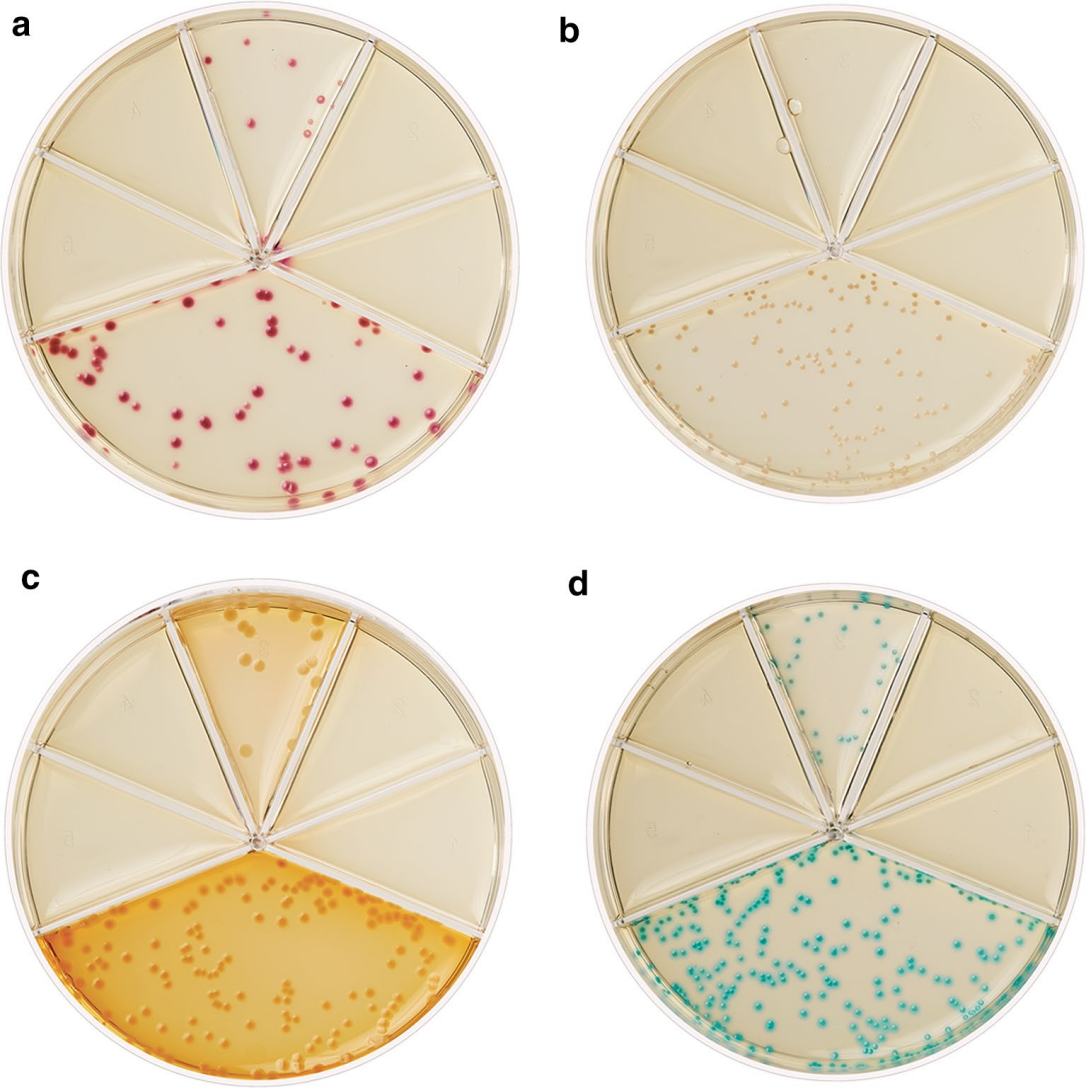
Bacterial identification by Flexicult^®^ Vet. The Flexicult^®^ Vet agar contains a chromogenic substrate to enhance bacterial identification, as colonies display *different colors* depending on the bacterial species. Examples of common pathogens involved in canine and feline UTI: *Escherichia coli* (**a**), *Staphylococcus pseudintermedius* (**b**), *Proteus mirabilis* (**c**), and *Enterococcus faecalis* (**d**)

Flexicult Vet A consists of a plastic Petri dish divided into one large compartment containing a non-selective growth medium (Mueller–Hinton BBL-II agar, Becton–Dickinson, Basel, Switzerland) supplemented with a chromogenic mixture, and five smaller compartments with the same medium supplemented with ampicillin (16 µg/ml), amoxicillin-clavulanate (12/6 µg/ml), cephalotin (12 µg/ml), enrofloxacin (0.5 µg/ml), and sulfamethoxazole-trimethoprim (38/2 µg/ml), respectively (Fig. 1). After flooding the plate with urine for 1–2 s, the excess of urine is discarded and the plate is incubated bottom-up at 35–37 °C for 24 h before reading. Bacterial loads are estimated by visual examination of colony density in the large compartment (Fig. 2), and strain susceptibility is deduced by growth in the smaller compartments containing antimicrobial concentrations based on Clinical Laboratory Standards Institute (CLSI) breakpoints [10]. As a secondary feature, the agar contains a chromogenic substrate to facilitate bacterial identification, as colonies display different colours depending on the bacterial species (Fig. 3).

### Field trial

Seven small animal veterinary clinics in Denmark, including one tertiary facility (The University Hospital for Companion Animals), one secondary facility (private referral hospital), and five smaller primary facilities participated in the study. Dogs and cats visiting these clinics between March and July 2013 were considered eligible for inclusion in the study when urine culture was part of their diagnostic work up. The clinics were supplied with Flexicult Vet A plates and incubators for in house testing, and the clinical staff members were instructed orally and in writing on how to use and interpret the test according to the manufacturer’s instructions. A portion of the urine samples was used by the clinical staff for in-house testing by Flexicult Vet A, and the remaining urine was submitted in sterile containers to the veterinary diagnostic microbiology laboratory at University of Copenhagen (Sund Vet Diagnostik, http://sundvetdiagnostik.ku.dk) or cultured overnight on Uricult^®^ dipslides (Orion Diagnostica, Nivå, Denmark) prior to submission to the laboratory. For each sample, the clinical staff member recorded the results obtained by Flexicult Vet A, including growth, bacterial concentration (CFU/ml), growth in the five antimicrobial compartments and bacterial identification. Data were also recorded about when and how the urine sample was collected (cystocentesis, urinary catheter or midstream catch), when the Flexicult Vet A plates were incubated and read, and who read them. Moreover, the clinical staff was asked to take pictures of the plates after incubation and to send them electronically to one of the investigators (SH) for interpretation.

At the diagnostic laboratory, ten µl of urine were cultured overnight at 37 °C on 5 % bovine blood agar for quantitative microbiology. Free catch samples were scored as positive (i.e. clinically relevant bacteriuria) on blood agar and on Flexicult Vet A if they contained ≥10^5^ CFU/ml, whereas the thresholds for urine collected by cystocentesis and catheter were ≥10^3^ and ≥10^4^, respectively. Isolates displaying distinct colony morphology were identified to the species level by MALDI-TOF mass spectrometry (MS) (VITEK^®^ MS, bioMérieux, Marcy l’Etoile, France), and their antimicrobial susceptibility profile was determined by broth microdilution using Sensititre^®^ COMPAN1F plates (Trek Diagnostic Systems, part of Thermo Fisher Scientific, East Grinstead, UK) according to CLSI [10]. The results of culture and susceptibility testing at the diagnostic laboratory were compared to those recorded by clinical staff and by the investigator using Flexicult Vet A.

### Optimization of antimicrobial panel

The composition of the antimicrobial panel of Flexicult Vet A was changed by replacing cephalotin with oxacillin for detection of MRSP. To optimize oxacillin concentration, plates containing the agar base supplemented with twofold dilutions of oxacillin (0.062–0.5 µg/ml) with or without 2 % NaCl were tested using 15 clinical MRSP isolates belonging to 10 multi-locus sequence types, and 15 randomly selected clinical *S. pseudintermedius* isolates susceptible to methicillin. Forty µl of bacterial suspensions containing 10^3^ and 10^4^ CFU/ml were spread on a quarter of each plate followed by incubation at 37 °C. Plates were read after 24 and 48 h. This work resulted in a new version of the product (Flexicult Vet B) with a compartment containing 0.125 ug/ml of oxacillin and 2 % NaCl.

### In vitro validation of Flexicult Vet B

The performance of Flexicult Vet B for susceptibility testing was validated in vitro using a collection of 110 clinical isolates identified to the species level by MALDI-TOF MS. The collection included 40 *E. coli*, 20 *S. pseudintermedius* (including 10 MRSP belonging to multi-locus sequence types ST71, ST267, ST269, ST270, ST271, ST272 and ST273), 12 *Proteus mirabilis*, 10 *Streptococcus canis*, 7 *Enterococcus faecalis*, 5 *S. aureus* (including 2 MRSA belonging to ST22 and ST239), 5 *Enterococcus faecium*, 4 *Enterobacter cloacae*, 3 *Pantoea agglomerans*, 2 *Klebsiella pneumoniae*, 1 *Klebsiella oxytoca*, and 1 *Enterobacter aerogenes*. Nine *E. coli* isolates were extended-spectrum β-lactamase (ESBL) producers with known genetic background: five producing CTX-M, two producing CMY and two producing both types of enzyme. These strains were included due to the clinical relevance of ESBL-producers in UTIs [6].

For each isolate, colonies from an overnight culture on 5 % bovine blood agar were suspended in saline to a turbidity of 0.5 McFarland (~10^8^ CFU/ml). The suspension was diluted to concentrations of approximately 10^3^ and 10^4^ CFU per ml. From each dilution, 300 µl was transferred to the large compartment of the Flexicult Vet B plate, and 100 µl to each of the small antimicrobial compartments, followed by gently tilting the plate and discarding excess fluid. Plates were read following overnight incubation at 35 °C. Strains growing in the antimicrobial compartments were regarded as resistant and their susceptibility profiles obtained by Flexicult Vet B were compared to those previously determined at the diagnostic laboratory by broth microdilution using the methodology described above [10].

### Statistical analysis

In the field trial, sensitivity and specificity of Flexicult Vet A for detection of clinically-relevant bacteriuria and for detection of resistance to the five drugs included in the test were estimated using laboratory culture on blood agar and MIC testing by broth micro-dilution as the reference standards, respectively. The performance of the test was evaluated according to the interpretations made by clinical staff and by the investigator. Sensitivity and specificity of the test for susceptibility testing were calculated by a 2 × 2 table using resistance (R) as a positive result (+) and susceptibility S as a negative (−) result. The Fisher exact test was used to evaluate whether clinically relevant bacteriuria detected by the reference standard was influenced by the method used for urine collection or by the time elapsed between sample collection and laboratory analysis. In the in vitro study, sensitivity and specificity of Flexicult Vet B for antimicrobial susceptibility testing were determined as described for the field trial.

## Results

### Field trial

A total of 72 urine samples were collected from 56 patients, including 42 (75 %) dogs and 14 (25 %) cats. For 13 patients (2 cats and 11 dogs) samples were collected in two or three occasions upon repeated admission of patients. For nine samples, Uricult^®^ dip slides were submitted to the laboratory after overnight culture. The remaining 63 samples were received and processed at the laboratory either on the day of collection (n = 47), the day after (n = 13), or two days after (n = 3). The majority of samples (61/72) were collected by cystocentesis, six by free catch and two by catheter. For three samples the sampling method was not reported.

Of the 72 samples, 25 (35 %) were culture-positive according to conventional culture in the diagnostic laboratory. Results from two of the culture-positive samples were excluded from further analyses because of failure of the diagnostic laboratory to report bacterial counts. The sensitivity (83 %, CI 0.63–0.93) and specificity (100 %, CI 0.92–1.00) of Flexicult Vet A for detection of clinically-relevant bacteriuria were the same regardless if the results were interpreted by clinical staff or by the investigator. Four false negative results were recorded by both the clinical staff and the investigator (Table 1). According to conventional culture, these samples were culture-positive with high bacterial concentrations (≥10^5^ CFU/ml), whereas on Flexicult Vet A two (positive for *E. coli* and *S. canis* by conventional culture) were sterile and the other two (positive for *P. mirabilis* and *E. faecium* by conventional culture) displayed bacterial growth below the defined threshold.

**Table 1:** Detection of clinically relevant bacteriuria by Flexicult Vet A in 70 urine samples from dogs or cats

	Reference standard		
	Positive	Negative	Total
Flexicult Vet A^a^			
Positive	19	0	19
Negative	4	47	51
Total	23	47	70

The interpretations by clinical staff and by the investigator are compared to the laboratory results obtained by aerobic culture on blood agar (reference standard)

^a^There were no differences in interpretation between clinical staff and investigator

Urine specimens collected by cystocentesis were more frequently culture-negative (68 %) compared to those collected by other methods (57 %) (Fisher exact test, one-tailed, *P* = 0.41). Among the 19 samples culture-positive by both conventional culture and Flexicult Vet A, two were excluded for evaluation of species identification due to failure of clinical staff to provide interpretation of species on Flexicult Vet A plates. Conventional culture of the remaining 17 samples resulted in growth of *E. coli* (n = 11), *K. pneumoniae* (n = 2), *P. mirabilis* (n = 1), *E. faecalis* (n = 1), *Pseudomonas aeruginosa* (n = 1) and *S. pseudintermedius* (n = 1). Clinical staff identified correctly the bacterial species in 9 (53 %, CI 0.31–0.74) samples. Identification mistakes occurred for all genera except *Enterococcus*. The investigator identified correctly the species in all the samples (100 %, CI 0.78–1.00).

The performance of Flexicult Vet A for susceptibility testing was evaluated using the laboratory results obtained by broth microdilution as the reference standard. False susceptibility was not observed for any of the 19 culture-positive samples for which the interpretations by clinical staff were available. The susceptibility results were correct for 70 and 76 % of the 94 drug-strain combinations tested when the results were interpreted by clinical staff and the investigator, respectively. The test was able to correctly detect all the strains that were resistant according to the reference standard method (100 % sensitivity, CI 0.72–1.00) but not all the strains that were susceptible [67 % specificity according to the clinicians’ interpretations (CI 0.56–0.76) and 73 % specificity according to the investigator’s interpretation (CI 0.62–0.82)]. False resistance to β-lactams (ampicillin, amoxicillin-clavulanate and cephalotin) was frequent, especially among *E. coli* isolates (Table 2).

**Table 2:** Antimicrobial susceptibility of *Escherichia coli* and other bacterial species in 19 culture-positive urine samples

Flexicult Vet A	Reference standard									
	AMP (R > 8 µg/ml)		AMC (R > 8/4^d^ µg/ml)		CEF^b^ (R > 4 µg/ml)		ENR^c^ (R > 2 µg/ml)		SXT (R > 2/38^e^ µg/ml)	
	R	S	R	S	R	S	R	S	R	S
*E. coli* (n = 12)										
R	2	8/7	0	9	0	8/7	0	0	0	0
S	0	2/3	0	3	0	4/5	0	12	0	12
Other (n = 7)^a^										
R	3	0	1	1/0	2	1/0	0	1/0	2	0
S	0	4	0	5/6	0	4/5	0	5/6	0	5

The interpretations of Flexicult Vet A by clinical staff and by the investigator are compared to the laboratory results obtained by broth microdilution (reference standard). A slash line is used to separate the results obtained by clinical staff (on the left) when they differed from those by the investigator (on the right)

*AMP* ampicillin, *AMC* amoxicillin-clavulanate, *CEF* cephalotin, *ENR* enrofloxacin, *SXT* trimethoprim-sulfamethoxazole

^a^Other species: *Proteus mirabilis* (n = 2), *Staphylococcus pseudintermedius* (n = 1), *Enterococcus faecalis* (n = 1), *Klebsiella pneumoniae* (n = 2), and *Pseudomonas aeruginosa* spp. (n = 1)

^b^Cefazolin was used instead of cephalotin for testing susceptibility to 1^st^ generation cephalosporins by the reference standard method

^c^One *Enterococcus* isolate intermediate to enrofloxacin according to the reference standard was not included in the analysis of enrofloxacin susceptibility

^d^The values 8 and 4 represent amoxicillin and clavulanate, respectively

^e^The values 2 and 38 represent trimethoprim and sulfamethoxazole, respectively

### Development and in vitro validation of Flexicult Vet B

Table 3 shows how MRSP detection was influenced by oxacillin concentration and presence of 2 % NaCl. Results were not affected by the inoculum size (10^3^ or 10^4^ CFU/ml). Inclusion of 0.125 µg/ml of oxacillin and 2 % NaCl in the Flexicult Vet B agar base resulted in the most reliable MRSP detection (100 % sensitivity and specificity) (Table 3).

**Table 3:** Growth of 15 methicillin-resistant (MRSP) and 15 methicillin-susceptible *Staphylococcus pseudintermedius* (MSSP) strains on Flexicult Vet agar base supplemented with different oxacillin concentrations (µg/ml) in the presence (+) or absence (–) of 2 % NaCl

Oxacillin conc. (µg/ml)	2 % NaCl	Incubation time (h)	MRSP growth/no. of isolates	MSSP growth/no. of isolates
0.5	–	24/48^a^	7/15	0/15
	+	24	9/15	0/15
	+	48	10/15	0/15
0.25	–	24/48^a^	10/15	0/15
	+	24	10/15	0/15
	+	48	11/15	0/15
0.125^b^	–	24/48^a^	13/15	0/15
	+	24/48^a^	15/15	0/15
0.062	–	24/48^a^	15/15	15/15
	+	24/48^a^	15/15	15/15

The results are presented as the proportions of correctly classified MRSP (growth) and MSSP (no growth) isolates following 24 and 48 h of incubation

^a^The same result was recorded after 24 and 48 h of incubation

^b^0.125 µg/ml oxacillin + 2 % NaCl was selected for Flexicult Vet B replacing the cephalotin compartment in Flexicult Vet A

**Fig. 4:**
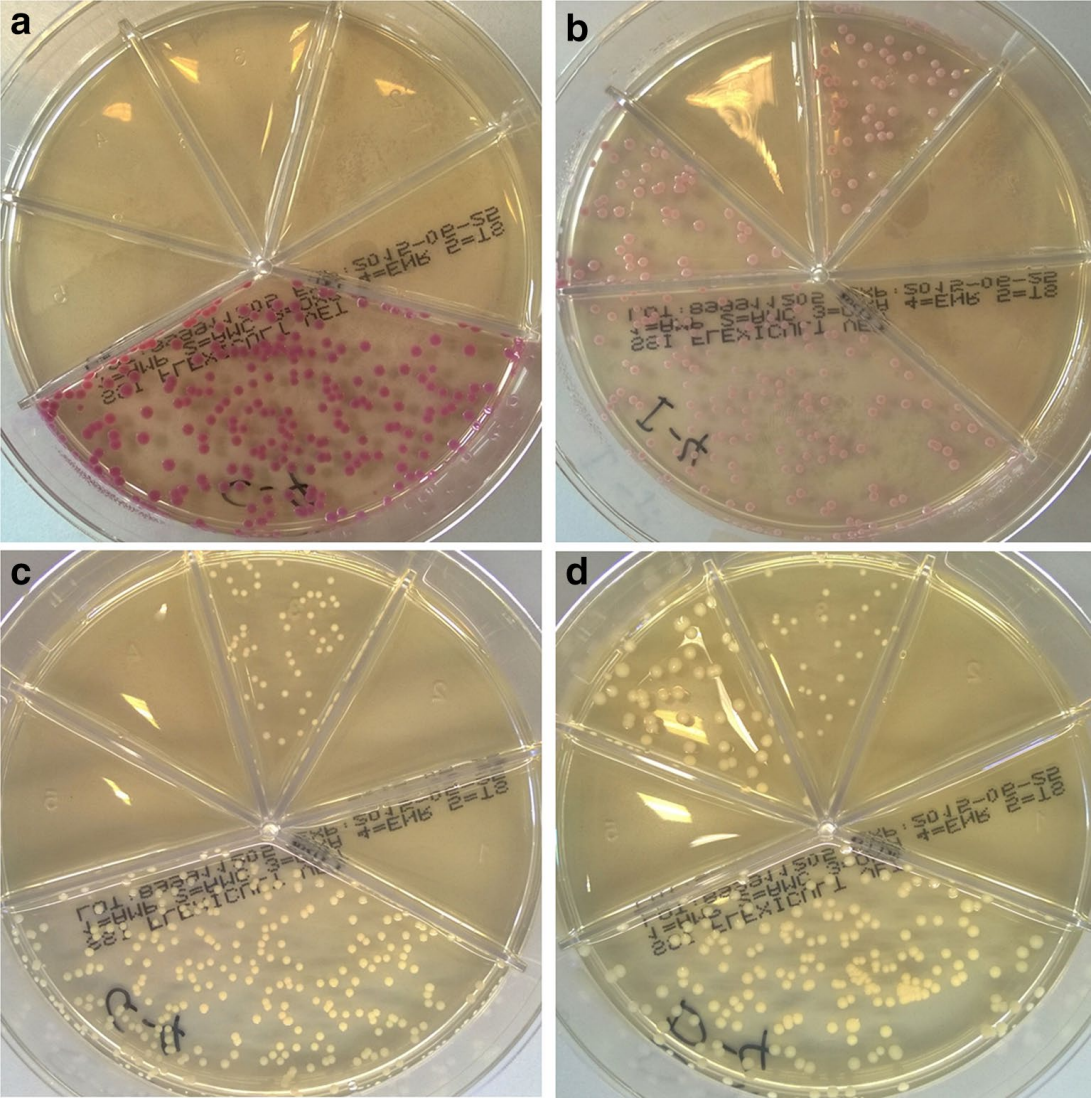
Colony appearance of *Staphylococcus pseudintermedius* and *Staphylococcus aureus* on Flexicult^®^ Vet plates. The two species are distinguishable after 48 h incubation since *Staphylococcus pseudintermedius* colonies are *pinkish* with a colour of variable intensity depending on the strain (**a**, **b**), whereas *Staphylococcus aureus* remained *white/yellow* (**c**, **d**)

The susceptibility results were correct for 94 % of the 465 drug-strain combinations tested by Flexicult Vet B (Table 4). The overall sensitivity (i.e. test’s ability to correctly detect resistant strains) and specificity (i.e. test’s ability to correctly detect susceptible strains) for antimicrobial susceptibility were 89 % (CI 0.83–0.94) and 96 % (CI 0.93–0.98), respectively. Eighty-seven (79 %) of the 110 strains’ susceptibility profiles were in full accordance with results from MIC determination by broth microdilution (gold standard). The overall error rate for the 465 antimicrobial-strain combinations tested was 6 % (Table 4). The most frequent error was false amoxicillin-clavulanate resistance in 12 of the 63 (19 %) *Enterobacteriaceae* isolates, including 10 *E. coli* (MIC = 8/4 µg/ml), four of which producing ESBL of the CTX-M type, one *P. mirabilis* (MIC = 8/4 µg/ml) and one *E. cloacae* (MIC ≤ 4/2 µg/ml). The second most common error was false ampicillin susceptibility in 3 of the 20 (15 %) *S. pseudintermedius* isolates. These three isolates were MRSP and had ampicillin MICs of 16 µg/ml (n = 1) and > 16 µg/ml (n = 2). Overall, only a single error was observed for enrofloxacin (false resistant *Proteus*) and for sulfamethoxazole with trimethoprim (false susceptible *Enterococcus*). Oxacillin susceptibility was correctly classified for all the 20 *S. pseudintermedius* tested, whereas one of the five *S. aureus* isolates was false resistant (Table 4). *S. aureus* and *S. pseudintermedius* were indistinguishable on Flexicult Vet B after 24 h incubation but after 48 h *S. pseudintermedius* colonies became pinkish with variable colour intensity among the strains, whereas *S. aureus* colonies remained uncoloured (Fig. 4).

**Table 4:** Comparison of the antimicrobial susceptibility results obtained for 105 clinical isolates by Flexicult Vet B and by the broth microdilution method (reference standard)

Bacterial species	Reference standard	Flexicult Vet B									
		AMP (R > 8 µg/ml)		AMC (R > 8/4^b^ µg/ml)		OXA^a^ (R > 0.25 µg/ml)		ENR (R > 2 µg/ml)		SXT (R > 2/38^c^ µg/ml)	
		R	S	R	S	R	S	R	S	R	S
*Escherichia coli* (n = 40)	R	22	–	5	–			13	–	18	–
	S	–	18	10	25			–	27	–	22
*Staphylococcus pseudintermedius* (n = 20)	R	3	3	1	2	10	–	5	–	8	–
	S	–	14	–	17	–	10	–	15	–	12
*Proteus mirabilis* (n = 12)	R	4	–	1	2			1	–	2	–
	S	–	8	1	8			1	10	–	10
*Enterococcus* spp. (n = 12)	R	2	2	2	2			8	–	2	1
	S	–	8	–	8			–	4	–	9
*Streptococcus canis* (n = 10)	R	–	–	–	–			1	–	–	–
	S	–	10	–	10			–	9	–	10
*Enterobacter spp.* (n = 8)	R	3	–	4	–			–	–	–	–
	S	1	4	1	3			–	8	–	8
*Staphylococcus*	R	1	–	1	–	2	–	2	–	1	–
*aureus* (n = 5)	S	–	4	–	4	1	2	–	3	–	4
*Klebsiella spp.* (n = 3)	R	2	1	–	–			–	–	–	–
	S	–	–	–	3			–	3	–	3
Total (n = 105)	R	37	6	14	6	12	–	30	–	31	1
	S	1	66	12	78	1	12	1	79	–	78

*AMP* ampicillin, *AMC* amoxicillin-clavulanate, *OXA* oxacillin, *ENR* enrofloxacin, *SXT* trimethoprim-sulfamethoxazole

^a^Oxacillin results were only interpreted for *S. pseudintermedius* and *S. aureus*, since this drug is a surrogate drug for detection of methicillin resistance in staphylococci

^b^The values 8 and 4 represent amoxicillin and clavulanate, respectively

^c^The values 2 and 38 represent trimethoprim and sulfamethoxazole, respectively

## Discussion

Rational antimicrobial use is a key element for control of antimicrobial resistance. Currently, various measures are being taken at the national, European and global level to reduce antimicrobial consumption in animals, including companion animals. Veterinary clinicians have the responsibility to implement these measures without impacting animal welfare. To enhance effective and sustainable implementation of rational antimicrobial use for treatment of UTIs, rapid and reliable point-of-care tests are needed to ensure that i) antimicrobials are prescribed/used only when necessary, and ii) the most appropriate drug is chosen taking into consideration the antimicrobial resistance profile of the causative strain. Our results show that the final product developed by this study (commercial name Flexicult^®^ Vet) is a useful point-of-care test to guide antimicrobial therapy of UTIs in small animals. The test provides overnight information on the presence of bacteria in urine and indicates which drug is appropriate for therapy. As such, it can be used to reduce empirical antimicrobial use and avoid unnecessary therapy. Compared to urine dipstick slides, it has the additional advantage of providing information on antimicrobial susceptibility. This is particularly important when resistance to the first tier drugs recommended by local or national antimicrobial guidelines is not infrequent. For example, amoxicillin is generally regarded as a first tier antimicrobial for treatment of uncomplicated lower UTIs in dogs and cats but resistance is relatively common in *E. coli* and other bacterial species isolated from these infections. In Denmark and Sweden, the prevalence of resistance to aminopenicillins (i.e. ampicillin and amoxicillin) in clinical *E. coli* isolates ranged from 16–26 % in 2011–2012 [7]. Thus, use of Flexicult^®^ Vet could potentially avoid prescription of amoxicillin for a large number of patients infected with strains resistant to this antibiotic.

The results of this study show good sensitivity and specificity of Flexicult^®^ Vet for both detection of clinically relevant bacteriuria and antimicrobial susceptibility testing. The results were generally concordant between clinical staff and the investigator, and between the investigator and the clinical microbiology laboratory (Tables 1, 2). Two notable exceptions were detected in the in vitro study: false amoxicillin-clavulanate resistance in *Enterobacteriaceae* and false ampicillin susceptibility in MRSP. These two errors should be regarded as major and very major errors, respectively [11]. A “major error” occurs when the new test indicates resistance in a strain that is categorized as susceptible by the reference method. This error reduces the range of antimicrobial options available to the clinician and may lead to unnecessary use of broad-spectrum drugs, with potential negative consequences on selection of resistance. A “very major error” occurs when a strain categorized as resistant by the reference method is reported as susceptible by the test. This type of error has a greater impact on patient care, since the clinician may choose a drug that is unlikely to be effective against the strain causing infection, with all the negative consequences of treatment failure. Based on this classification, false ampicillin susceptibility in MRSP appears to be the most important problem of the test. However, this error was solved by the inclusion of oxacillin in the test, since all the isolates displaying false ampicillin susceptibility were resistant to oxacillin and should be categorized as resistant to all veterinary β-lactams according to international standards [10]. Thus, this error is irrelevant if clinical staff is trained to identify staphylococcal strains and interpret the oxacillin susceptibility result correctly. It should be noted that the oxacillin concentration was chosen based on the clinical breakpoint for *S. pseudintermedius* (R ≥ 0.5 µg/ml), which is eightfold lower than for *S. aureus* (R ≥ 4 µg/ml) [10]. This explains why false oxacillin resistance was detected in one methicillin-susceptible *S. aureus* (Table 4). As the colony appearance becomes distinguishable between the two species after 48 h incubation (Fig. 4), we recommend that incubation of Flexicult^®^ plates is extended to 48 h when suspected staphylococcal colonies are detected in the oxacillin compartment. Moreover, as a matter of principle, presumptive MRSP and MRSA should be confirmed and subjected to antimicrobial susceptibility testing by a diagnostic laboratory to guide antimicrobial choice.

In relation to the false amoxicillin-clavulanate resistance observed in *Enterobacteriaceae*, it should be noted that the MIC of amoxicillin-clavulanate (8/4 µg/ml) was just below the clinical breakpoint (R > 8/4 µg/ml) in nine out of the 10 false resistant isolates, and five of them were CTX-M-producing *E. coli*. Some studies in human medicine suggest that amoxicillin-clavulanate might be considered as a second-line agent for management of lower UTI caused by ESBL producers with even higher MIC than that observed in these isolates [12]. Clinical cure is likely due to the high drug concentrations achieved in urine and the theoretical inactivation of ESBLs by clavulanate. However this is a controversial issue and neither retrospective nor prospective studies have been performed to provide evidence of clinical cure in small animals. We recommend that Flexicult^®^ Vet plates are submitted to a diagnostic laboratory and specialist advice is sought when growth is observed in all the five antimicrobial compartments. Furthermore, instructions to users should be made available online to reduce the risk of errors in the interpretation of the antimicrobial susceptibility results, for example by providing detailed guidelines on how to interpret growth of one or few colonies in one of the antimicrobial-containing fields.

In the field study, pathogen identification by clinical staff was unreliable, since the species was correctly identified in only 53 % of the culture-positive samples. Similar findings have been reported for dipstick slides in both human and veterinary settings [13–15]. Although pathogen identification may be regarded as a secondary feature of the test as it provides information of limited clinical relevance, particular attention should be given to avoid certain errors that may lead to inappropriate antimicrobial choice. For example, misidentification of staphylococci may cause erroneous prescription of β-lactams for treatment of MRSP and MRSA UTIs despite growth in the oxacillin compartment, which is indicative of resistance to all β-lactams for this bacterial group. Our study shows that the charts provided by the manufacturer may not be sufficient to avoid frequent errors in pathogen identification. Importantly, our results also show that interpretation can be improved significantly with experience, as indicated by the excellent score obtained by the investigator. Accordingly, the authors recommend that training sessions are offered by the manufacturer to enhance correct interpretation of the results, including examples of mixed cultures, which make identification more difficult compared to pure bacterial cultures. Users are also recommended to validate their interpretive skills by sending plates regularly to a diagnostic laboratory for species identification.

Notably, the use of true urine samples instead of pure cultures suspended in saline seems also to reduce the performance of the test for antimicrobial susceptibility testing under real-life conditions, as indicated by the lower specificity estimated in the field study (73 %) compared to the in vitro study (95 %) based on the investigator’s interpretations. This discrepancy was largely due to high rate of false resistance to β-lactams observed for *E. coli* in the field study. The reason for this discrepancy remains unknown but it appears that urine may interfere with the results of β-lactam susceptibility testing as this problem was not observed when the plates were inoculated with bacterial suspensions in sterile saline in the in vitro study.

Due to the circumstances described in the results section, only 17 and 19 culture-positive samples were evaluated for bacterial species identification and antimicrobial susceptibility testing in the field trial, respectively. This is a major limitation of the study, since the performance of a point-of-care test like this should primarily be evaluated based on the results obtained by clinical staff using urine samples. Thus, further studies are warranted to evaluate the performance of the test in the field. The high (65 %) proportion of culture-negative samples in the field trial may in part be explained by the participation of tertiary and secondary facilities in the study and by the inclusion criteria being any indication for culture, thus not restricted to patients displaying symptoms of UTI. An even lower percentage of 17.5 % positivity was recently reported among 5923 urine samples cultured in a UK tertiary referral hospital between 1999 and 2009 [16]. Culture-negative results are particularly common when screening animals with low urinary specific gravity for UTI [17], and for samples collected by cystocentesis [15], as most samples (61/72) used in this study. The uncertainty of bacterial infection and the consequent risk of antimicrobial overuse in patients with suspected UTI highlight the usefulness of point-of-care tests allowing clinicians to take evidence-based decisions on whether antimicrobial therapy is needed. However, responsible use of culture-based point-of-care tests requires trained staff and adequate laboratory facilities and waste management procedures.

## Conclusions

Flexicult^®^ Vet is a time- and cost-effective point-of-care test for detection of bacteriuria and antimicrobial susceptibility testing of uropathogens in small animal veterinary clinics that meet the minimal requirements for in-house culture. Even though we identified important shortcomings regarding species identification by clinical staff and β-lactam susceptibility testing of *E. coli*, rational use of this product may guide antimicrobial choice and facilitate implementation of the current antimicrobial use guidelines for treatment of UTIs. Following this study the manufacturer has revised instruction manuals illustrating interpretation of bacterial counts and species. Apart from this initiative we recommend adequate training of clinicians to reduce the risk of interpretative errors of potential impact on patient care, such as those regarding staphylococcal identification and growth in the oxacillin-containing compartment.

## Abbreviations

ESBL: extended-spectrum β-lactamase
MIC: minimum inhibitory concentration
MRSA: methicillin-resistant *Staphylococcus aureus*
MRSP: methicillin-resistant *Staphylococcus pseudintermedius*
UTI: urinary tract infection
